# Misconduct in scientific publications

**DOI:** 10.1590/2177-6709.23.3.007-008.edt

**Published:** 2018

**Authors:** Sigmar de Mello Rode, Rodrigo Rios Faria de Oliveira, Luiz Renato Paranhos

**Affiliations:** 1 Universidade Estadual Paulista, Instituto de Ciência e Tecnologia de São José dos Campos, Curso de Odontologia (São José dos Campos/SP, Brazil).; 2 Faculdades Integradas ASMEC, Curso de Direito (Pouso Alegre/MG, Brazil).; 3 Universidade do Vale do Sapucaí, Curso de Ciências Contábeis (Pouso Alegre/MG, Brazil).; 4 Universidade Federal de Uberlândia, Faculdade de Odontologia (Uberlândia/MG, Brazil).

According to the Council of Science Editors^1^, the term “research misconduct” applies to any situation presenting either inadequate treatment of the individuals involved in the research or intentional manipulation of the scientific records so they will not reflect the truth. Even though this is fairly easy to conceptualize, it is often difficult to identify, especially in scientific publications.

When reporting the “inadequate treatment of the individual”, a more favorable situation is perceived, considering the need for submitting the studies to organizations that regulate the bioethical issues involving animals, human beings, or the environment. When identifying misconduct, it is important to verify whether it was intentional or “accidental”, whereas both deserve some type of penalty. Reports and penalties for research misconduct have been increasingly exposed, and they may appear as a warning, text revision, or even layoffs and fines.

There are several types of misconduct in research reports. Among the most serious ones are forgery (alteration/misrepresentation) or the fabrication of data and images, because they detract from the truth and lead to false premises, confusing clinical professionals and/or researchers.

The partial or full copy of a text, unauthorized or unreferenced, characterizes plagiarism. Perhaps this is one of the main problems found in publications, and it is serious to the point of causing several education, research, and development institutions to create codes of instruction and conduct, such as the Research Support Foundation of the State of São Paulo, Brazil (FAPESP)^2^ and the National Counsel of Technological and Scientific Development (CNPQ)^3^, among others. Tools to identify plagiarism are constantly created, but they only identify similarities; therefore, there is no “magic number” to characterize this misconduct, because the similarities depend on careful interpretation.

The legal system in Brazil states that plagiarism is, in the terms of article 184 of the Criminal Code^4^, the violation of copyrights and other associated rights. It is also necessary to verify article 5^th^, XXVII of the Constitution of the Brazilian Republic^5^, which section states that *“authors hold the exclusive right of use, publication, or reproduction of their work, transferable to the heirs for the time determined by the law.”* The copyright legislation is hereby verified only in order to complement the issue under analysis, in Law 9610/1998^6^, which affirms that authors hold *“the moral and property right over the work they created”.*

Thus, it is easy to realize that authors hold the inherent rights over their creations and, in the case of self-plagiarism, which is an element that is not typified in the Brazilian legislation, they would be considered simultaneously victim and offender. Therefore, it may be affirmed that some authors report the definition of “self-plagiarism” incorrectly. Ultimately, a potential accountability might be conceived in the civil context regarding the assignment of copyrights to third parties.

The authorship of the publications is also a significant problem, because authors do not often standardize the citation of authors in the text, either placing incorrect authors or neglecting to insert them. The health field relies on the norms of the International Committee of Medical Journal Editors (ICMJE)^7^, which recommends that the author of a scientific text should have a substantial contribution to the creation and design of the scientific work, as well as to the collection, interpretation, and analysis of data; participate in the writing and critical review of the work with an actual intellectual contribution to the content; and approve the final content for publication. In case these conditions are not met, the author should be cited in the Acknowledgments section. 

Unfortunately, it is common to hear reports of pressure from coordinators, professors, and advisers who compel the subordinates - mostly undergraduate and graduate students - to cite the names of people who most often are not even aware of the content of the article to be published. Thus, influence and “gratitude” do not determine the authorship of a scientific article^8^. 

According to Rode and Galletti Queiroz,^9^ *“the relationship between science and scientific output is evident: the latter being the product of the former, science is done by conducting ethical studies and then disseminated by publication of technical-scientific articles.”* Hence, honesty is essential for authors and ignorance can no longer be clai med. As the popular saying goes, *“Trust is like paper, once it is crumpled it can never be perfect again.”*

Good readings!

Sigmar de Mello Rode, Rodrigo Rios Faria de Oliveira, Luiz Renato Paranhos
